# Model Updating Concept Using Bridge Weigh-in-Motion Data

**DOI:** 10.3390/s23042067

**Published:** 2023-02-12

**Authors:** Doron Hekič, Andrej Anžlin, Maja Kreslin, Aleš Žnidarič, Peter Češarek

**Affiliations:** 1 Faculty of Civil and Geodetic Engineering, University of Ljubljana, Jamova cesta 2, 1000 Ljubljana, Slovenia; 2 Department of Structures, Slovenian National Building and Civil Engineering Institute, Dimičeva ulica 12, 1000 Ljubljana, Slovenia

**Keywords:** monitoring, bridge, viaduct, bridge weigh-in-motion (B-WIM), structural health monitoring (SHM), finite element (FE), calibration, model updating

## Abstract

Finite element (FE) model updating of bridges is based on the measured modal parameters and less frequently on the measured structural response under a known load. Until recently, the FE model updating did not consider strain measurements from sensors installed for weighing vehicles with bridge weigh-in-motion (B-WIM) systems. A 50-year-old multi-span concrete highway viaduct, renovated between 2017 and 2019, was equipped with continuous monitoring system with over 200 sensors, and a B-WIM system. In the most heavily instrumented span, the maximum measured longitudinal strains induced by the full-speed calibration vehicle passages were compared with the modelled strains. Based on the sensitivity study results, three variables that affected its overall stiffness were updated: Young’s modulus adjustment factor of all structural elements, and two anchorage reduction factors that considered the interaction between the superstructure and non-structural elements. The analysis confirmed the importance of the initial manual FE model updating to correctly reflect the non-structural elements during the automatic nonlinear optimisation. It also demonstrated a successful use of pseudo-static B-WIM loading data during the model updating process and the potential to extend the proposed approach to using random B-WIM-weighed vehicles for FE model updating and long-term monitoring of structural parameters and load-dependent phenomena.

## 1. Introduction

In 2021, total road freight transport in terms of tonne–kilometres in the European Union (EU) increased by 7% compared to 2020 [1]. The EU’s new stream of investment into sustainable, safe, and efficient transport infrastructure will raise the need for efficient management of road infrastructure [2]. The lifespan of bridges is increasingly coming to an end, also boosted by the expected impact of climate change on the increased number and severity of temperature cycles, wind, and flood conditions [3]. With that, the challenge of maintaining bridge performance at the desired level becomes more significant. Bridges can be evaluated using the COST Action 1406 [4] or the Long Bridge Performance Program [5] methodologies, which define four key performance indicators [6]: Functionality, Costs, Structural Condition, and Structural Integrity. All these factors can be addressed by directly or indirectly integrating permanent structural health monitoring (SHM) system results.

Maintaining full bridge functionality requires adequate monitoring of structural integrity and its condition. By tailoring the scope of monitoring to the infrastructure’s needs and importance in the road network, data can be collected and processed to support smart decisions, including the costs that sustain the bridge at the desired operational level. For large civil engineering structures, the impact of up-to-date data on their performance often outweighs the cost of design, setup, and maintenance of a comprehensive SHM system [7]. On the other hand, SHM systems are rarely installed on less important structures due to the relatively high costs for bridge owners/stakeholders. Solutions, such as population-based SHM [8], are exciting but unusual alternatives. Taking advantage of the systems already installed on the bridges and upgrading them with monitoring capabilities could increase interest in using SHM on less important structures.

A measuring device with such potential is a bridge weigh-in-motion (B-WIM) system. Initially developed by Moses in 1979 [9], B-WIM systems were and still are mainly used to weigh the freight traffic in motion. However, years of development have upgraded B-WIM systems to use bridges not only as large scales but also to measure structural responses through time to calculate the performance indicators [10], like influence lines, girder distribution factors, and dynamic amplification factors. By deducting the temperature impact, bridge engineers can extract vital information to support and optimise bridge reliability calculations. B-WIM-based systems can provide a cost-efficient SHM solution for smaller, less critical bridges for which allocating traditional monitoring expenses is unrealistic. Bridge instrumentation can be used for a dual purpose, to measure the traffic load spectrum and bridge performance under individual vehicle crossings. The bridge also need not be closed during instrumentation and measurements, which is an important advantage on heavily trafficked roads. Therefore, if a bridge is already instrumented for vehicle weighing, it would be beneficial if the same data could be used for SHM.

Finite element (FE) model updating has not yet been performed with the data from B-WIM sensors. The finite element method (FEM) is the standard tool for modelling structural behaviour. However, due to a wide range of simplifying assumptions that rely on engineering judgment related to boundary conditions and uncertainties in material characteristics, the FE model only approximates the actual structure. There is, therefore, always a need to improve the initial FE model [11]. FE model updating, sometimes referred to as calibration or correction, is viable if the behaviour of the structure is known, i.e., measured.

The objective functions minimise the difference between the responses of the actual structure and the model. Minimising can be performed by manually changing the following variables: structure’s material parameters, boundary conditions, etc., and/or by automatic nonlinear optimisation, where updated variables follow the optimisation algorithm. Generally, two types of measurements are performed on bridges: (1) dynamic characteristics measurements or dynamic SHM, and (2) structural response measurements during a static load test or static SHM. Studies such as [12] that consider both types of measurements are scarce.

In dynamic characteristics measurements or vibration-based bridge health monitoring, the structural response is typically measured with accelerometers [13,14,15] and sometimes with strain measurement devices [16,17,18]. Several methods were developed to extract the modal parameters (natural frequencies, mode shapes, etc.) from vibration-based bridge health monitoring. Examples are the basic frequency domain (BFD) method, frequency domain decomposition (FDD), enhanced frequency domain decomposition (EFDD), and stochastic subspace identification (SSI), described in more detail in [19,20].

Fewer model updating studies are based on static than dynamic SHM since road closure during static SHM, and the corresponding traffic interruption costs, can be significant. Dynamic SHM is generally less costly and can be installed faster than static SHM. The load can be unknown since the modal parameters are derived from the ambient or free vibration of the structure and can be evaluated continuously. However, it was shown in [12,21,22] that the updated FE model based on dynamic SHM measurements (natural frequencies) does not necessarily match the updated FE model based on static SHM measurements. Load tests—either diagnostic, proof, or soft [23]—are therefore significant and necessary addition to the dynamic SHM measurements when a generally valid and accurate FE model is sought [12]. In particular, if the FE model is updated based on the dynamic SHM measurements and afterwards used to simulate traffic loads. Such an FE model can overestimate bending stiffness as ambient vibrations during dynamic SHM measurement are often too low to excite some structural elements, such as sliding bearings, pins, and expansion joints [24].

This paper investigates the possibility of using data from sensors installed primarily for the B-WIM purpose for model updating. Most measure longitudinal strains and are installed around the mid-span of the superstructure. Strain time histories are not captured continuously but only when freight vehicles cross the bridge. Moreover, vehicles pass the superstructure at full speed, which differs from conventional static SHM measurements, where the load is positioned statically at known positions. Such B-WIM SHM extension would enable continuous updating of the digital twin—the FE model of the actual structure—based on the long-term response to heavy traffic, thus providing insights into its structural health over time.

The paper is organised as follows: Section 2.1 presents a case study viaduct. Section 2.2.1 describes extensive long-term monitoring of a viaduct, focusing on the longitudinal strain measurements of the most heavily instrumented span P14D. Section 2.2.2 presents the calibration vehicle results obtained as a part of the B-WIM system calibration and used for FE model updating. Section 2.3 presents the structural system and FE model of the considered span. Section 2.4 deals with the objective function definition, manual FE model refinement procedure, and algorithms for automatic nonlinear optimisation. Results are presented in two parts. Section 3.1 describes the selection of the variables to be updated and the determination of the vehicle position that causes the greatest response. Section 3.2 shows the updated FE variables.

## 2. Materials and Methods

### 2.1. History of the Viaduct

**Figure 1:**
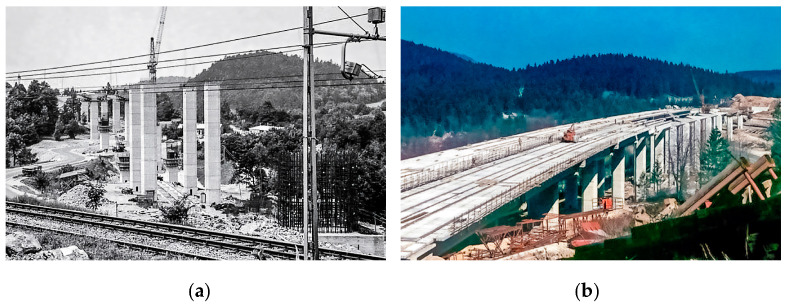
Construction of the (**a**) Piers [25] and (**b**) Superstructure [26].

The case study is a twin multi-span precast I girder type viaduct (in the following designated as Ravbarkomanda viaduct), with an overall length of 588 m and 544 m. It is located on the heavily trafficked 5th Trans-European corridor from Venice in Italy to Lviv in Ukraine. It crosses a double-track railway line and a state road twice. Constructed 50 years ago (Figure 1), in the early days of precast prestressed concrete construction and with inadequate quality control of the construction work, it suffered from severe deterioration accelerated by harsh winter conditions and massive use of de-icing salt. Four major rehabilitations were applied, about one every ten years.

The construction finished in 1972 when the seismic design rules, compared to today’s principles [27], underestimated earthquake forces and the structure’s non-ductile performance, mainly exhibited in the construction details of the hollow piers [28].

**Figure 2:**
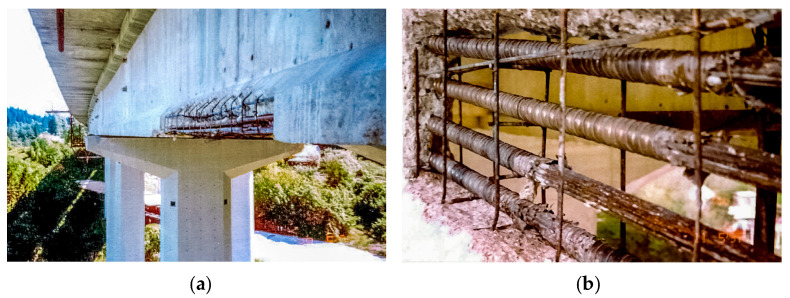
(**a**) Corroded prestressing tendons in I-shaped precast concrete girders of the viaduct deck; (**b**) Detailed view of the corroded prestressing tendons.

The viaduct was first repaired in the late 80s, followed by complete viaduct deck rehabilitation in the second half of the 90s due to the corroded prestressing tendons in precast reinforced concrete girders of the selected spans (Figure 2).

At the beginning of the new millennium, extensive analytical and experimental studies revealed the insufficient shear capacity of short piers due to poor seismic design. For this reason, in 2008, seismic rehabilitation of the viaduct piers started, resulting in an additional concrete jacket in the region of plastic hinges. However, durability issues were still not thoroughly addressed. Finally, in 2017, a comprehensive reconstruction of the viaduct rehabilitated or reconstructed all elements of the viaduct, including its equipment. As part of the reconstruction, a permanent remote monitoring system was installed [29].

### 2.2. Establishment of the Monitoring System

Increasing development and deployment of SHM systems is limited particularly to large newly built bridges [30], such as Runyang Suspension Bridge, which was, upon its completion in 2005, the longest suspension bridge span in China [31]. All long-term SHM applications, however, are not limited to landscape bridges. Applications on less striking bridges can be found as well, such as the long-term SHM of a strengthened railway bridge in France [32], bowstring-arch railway bridge in Portugal [33], and concrete cable-stayed bridge in Portugal [34].

The motivation for establishing the monitoring system on the viaduct was primarily its age and condition. In addition, the daily number of heavy goods vehicles over 7.5 tonnes increased from 3300 before joining the European Union in 2004 to over 8800 in 2021, with the prospect of a further substantial rise in the coming years. Consequently, during the latest reconstruction, the viaduct owner decided to increase its load-bearing capacity with carbon fibre rods in the viaduct deck.

To follow the performance of a structure that had corroded (internal) prestressing tendons replaced with external prestressing tendons on the selected spans in the 90s and its deck widened in 2017, the owner decided to permanently monitor some critical performance parameters. To achieve this, 216 sensors measure longitudinal strains on the main girders, caps on top of the piers, and the newly constructed deck extensions, plus the temperature and the vibration of the external tendons to determine the possible change in their natural frequencies and the related tensioning force. Simultaneously, the B-WIM system uses longitudinal strain measurements on the main girders for weighing vehicles.

#### 2.2.1. Sensor Description

**Figure 3:**
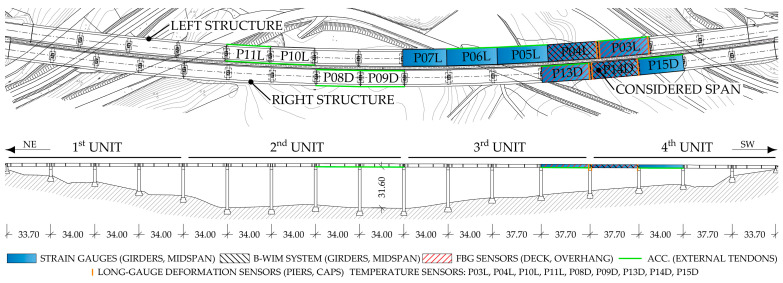
Plan and side views of the Ravbarkomanda viaduct with a schematic view of sensor types and locations.

**Figure 4:**
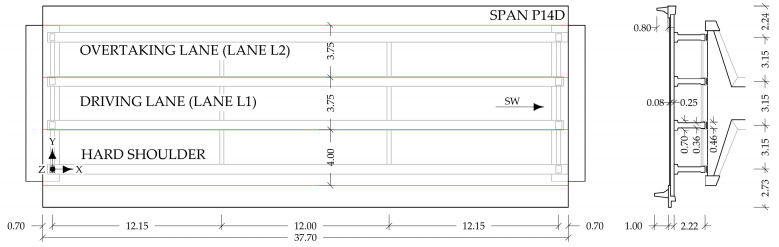
The dimensions of the span P14D.

The viaduct consists of two parallel structures: the right structure carries the traffic from the northeast (Ljubljana direction), and the left structure in the opposite direction. Monitoring is established on both structures, as shown in Figure 3. Longitudinal strains on the main girders are measured in the spans P03L, P04L, P05L, P06L, P07L, P13D, P14D, and P15D. Some sensors in the spans P04L and P14D are devoted to the B-WIM. The FE model updating presented in this paper is performed only for the span P14D, the dimensions of which are shown in Figure 4. Strains on the carbon fibre rods are measured in the spans P03L, P04L, P13D, and P14D. Accelerations of the external prestressing tendons, which are strengthening the main girders, are acquired in the spans P03L, P05L, P06L, P10L, P11L, P08D, P09D, P13D, and P15D. Temperature is measured in spans P03L, P04L, P10L, P11L, P08D, P09D, P13D, P14D, and P15D.

**Figure 5:**
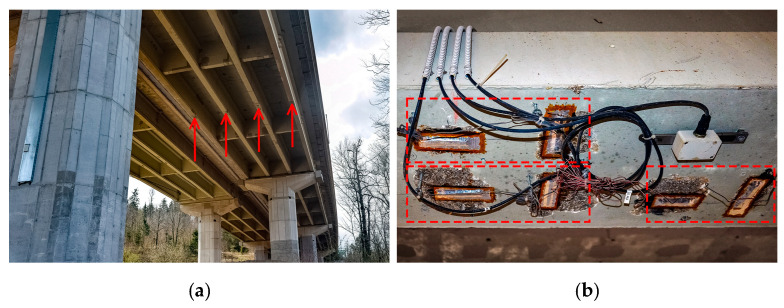
Strain gauges near mid-span of the main girders (span P14D): (**a**) Distance view with arrows denoting the strain gauge locations on each girder; (**b**) Close-up view of three measurement points near the mid-span of a selected main girder in P14D.

Each span consists of four main girders. Their response is monitored from the longitudinal strain measurements near mid-spans with 120 and 350-Ohm half-Wheatstone bridge strain gauges, as shown in Figure 5. The number of strain gauges in each span varies as follows:

- P05L, P06L, P07L: one strain gauge per each external main girder (6 overall);
- P03L, P13D, and P15D: one strain gauge per each main girder (12 overall);
- P14D: three strain gauges per each main girder (12 overall);
- P04L: four strain gauges per main girder (16 overall).

The strain time history data during the free traffic flow are recorded with 500 samples per second and filtered with a 35 Hz low pass filter. For further analysis, the data of each vehicle loading event are processed and stored as statistical blocks containing the minimum, maximum, and mean value of the strain. These values are related to those measured at the establishment of the monitoring system at the end of 2017. An event, in addition to vehicle data, contains measured strains during the vehicle passage. Its recording is triggered when strain levels exceed the idle state, corresponding to the bridge with no vehicle on it, for at least 2 μm/m. 

**Figure 6:**
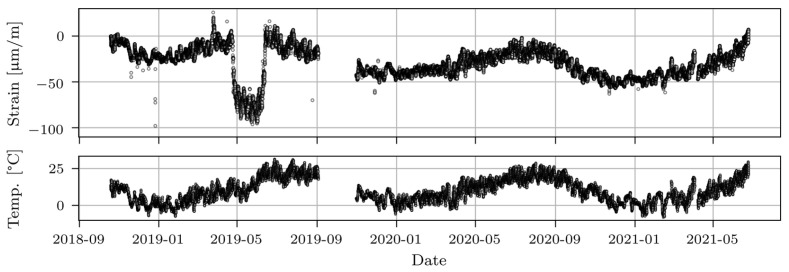
Hourly averages of longitudinal strains measured by strain gauge sensor in span P14D with eliminated traffic loading effect (upper part) and hourly temperature averages (lower part).

Figure 6 (upper part) shows the main girder longitudinal strains measured by a strain gauge sensor in span P14D, and the corresponding temperature (lower part) for the period from October 2018 and June 2021. The dots present the hourly average minimum strain values from all events captured in one hour. In such a way, the influence of traffic on the viaduct response was eliminated. In general, measured strains follow the temperature variation except in May 2019, during asphalt removal, which changed the stiffness and mass of the structure. The monitoring system successfully detected these activities. The data absence in September 2019 and October 2019 was due to the absence of power during viaduct renovation.

**Figure 7:**
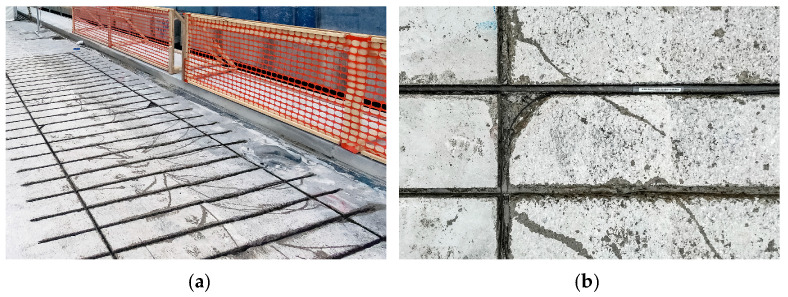
FBG sensors on the carbon fibre rods (span P13D): (**a**) Distance view; (**b**) Close-up view.

The overhangs of the widened deck were reinforced with carbon fibre rods, as shown in Figure 7. Their long-term behaviour is monitored by 40 Fiber Bragg Grating (FBG) sensors on the carbon fibre rods. The strain time history is recorded with one sample per second, and only statistical blocks (minimum, maximum, and mean values) of all 1-min long strain time domain signals are stored.

**Figure 8:**
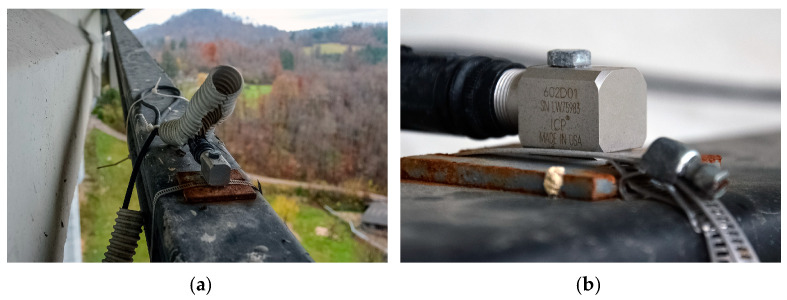
Accelerometers on the external prestressing tendons: (**a**) Distance view; (**b**) Close-up view.

The external prestressing tendon monitoring is based on natural frequencies derived from one-hour acceleration records. The single axial accelerometers (Figure 8) are installed in 4 spans at 12 locations on the right viaduct and in 5 spans at 12 locations on the left viaduct. The acceleration records in the time domain are recorded with 500 samples per second. Fast Fourier Transformation is used to calculate the natural frequencies of the tendons. To indirectly monitor the force in the prestressing tendons from the identified natural frequencies, the principle based on the string equation [35] is used.

**Figure 9:**
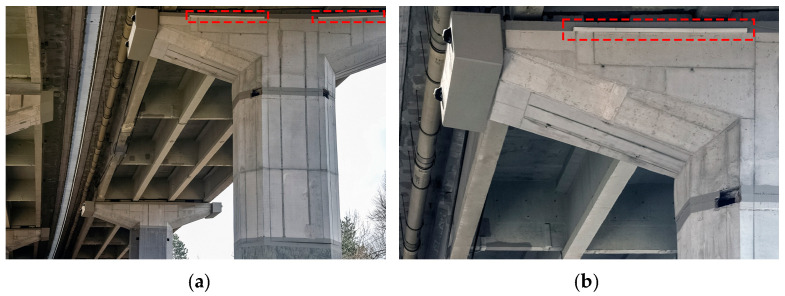
Long gauge deformation sensors (marked with red dashed line) on the pier: (**a**) Distance view; (**b**) Close up view.

The pier caps are monitored with long gauge deformation sensors installed at six locations on both viaducts (Figure 9). Displacements in 5-min intervals are stored and normalised to the length of the sensor to obtain the strains.

#### 2.2.2. Calibration Vehicle Passages

**Figure 10:**
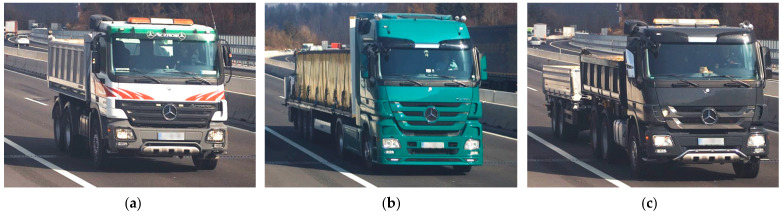
Static pre-weighted bridge weigh-in-motion (B-WIM) system calibration vehicles in lane L1: (**a**) V1; (**b**) V2; (**c**) V3.

The B-WIM systems are calibrated with vehicles with known axle loads and distances. For the considered viaduct, three calibration vehicles were used. Each passed both viaducts 20 times on the driving lane (lane L1) and 20 times on the overtaking lane (lane L2). Overall, 60 passages per lane were recorded on each viaduct. However, some were not considered due to the simultaneous presence of another vehicle on the viaduct and/or due to excessive deviation in the transverse position. Finally, 16, 17, and 18 passages of vehicles V1, V2, and V3, respectively, were considered in lane L1, and 20, 13, and 20 passages in lane L2. Calibration vehicles driving in lane L1 are shown in Figure 10. Their axle loads and spacings are given in Table 1. Only L1 passages are considered in this paper.

**Figure 11:**
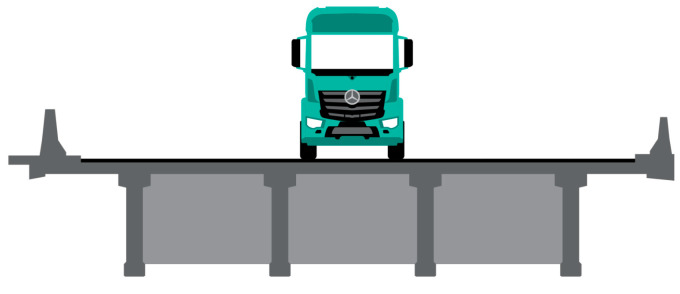
The transverse position of the calibration vehicle in lane L1.

The calibration vehicle in lane L1 is positioned approximately in the centreline between the two internal longitudinal girders (Figure 11).

**Figure 12:**
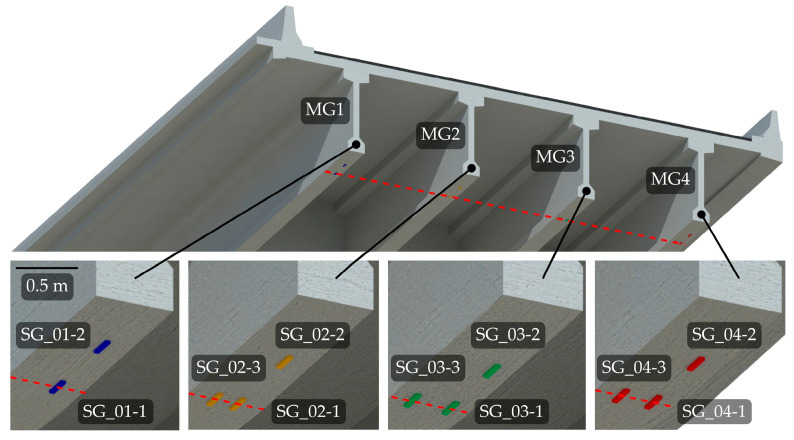
Locations and labels of the strain gauges in the span P14D with red dashed line denoting the mid-span.

This paper only considers model updating of the span P14D, as explained in Section 2.2.1. Figure 12 shows the disposition of all strain gauges that were considered in this study, together with their labels and labels of the main girders. One of the initially installed sensors on the MG1 was identified as corrupted and was omitted from the analysis (11 instead of 12 sensors were considered). Sensors are located near the mid-span indicated with a red dashed line in Figure 12. Deviation from the mid-span in the longitudinal direction is less than 60 cm (less than 2% of the span length). The transverse position of the strain gauges also deviates from girder to girder: for MG1 and MG2, each strain gauge is mounted 16 cm from the nearest outer face, for MG3 12 cm and MG4 14 cm, respectively.

**Figure 13:**
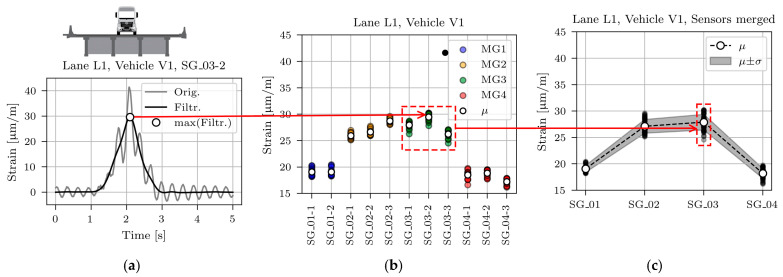
Process of determining the mean (*μ)* and standard deviation (*σ*) for sensors SG_01, SG_02, SG_03, and SG_04 for vehicle V1: (**a**) Time domain signal; (**b**) Maximum values for all passages and individual sensors; (**c**) Maximum values for all passages and merged sensors.

**Figure 14:**
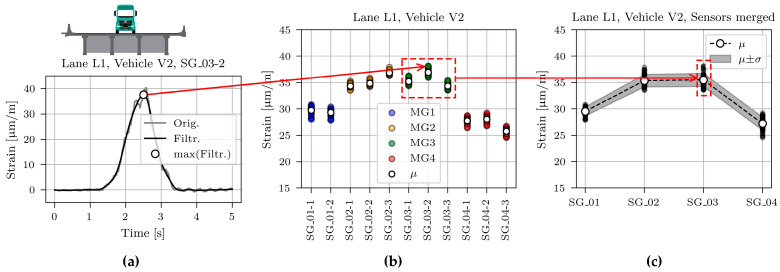
Process of determining the mean (*μ)* and standard deviation (*σ*) for sensors SG_01, SG_02, SG_03, and SG_04 for vehicle V2: (**a**) Time domain signal; (**b**) Maximum values for all passages and individual sensors; (**c**) Maximum values for all passages and merged sensors.

**Figure 15:**
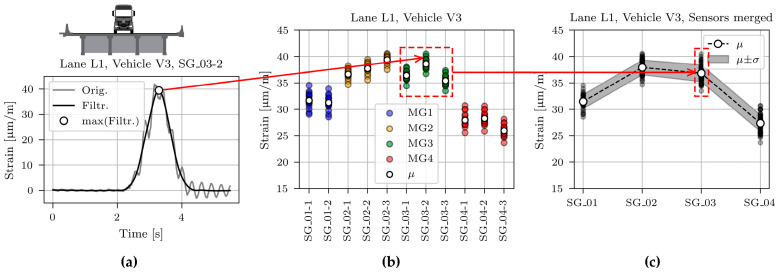
Process of determining the mean (*μ)* and standard deviation (*σ*) for sensors SG_01, SG_02, SG_03, and SG_04 for vehicle V3: (**a**) Time domain signal; (**b**) Maximum values for all passages and individual sensors; (**c**) Maximum values for all passages and merged sensors.

Figure 13a, Figure 14a and Figure 15a show typical time domain signals for sensor SG_03-2, caused by all three calibration vehicles driving in lane L1. The grey line denotes non-filtered, and the black line processed signals with the 2 Hz low pass filter to eliminate the dynamic component of the signal. The basis for determination of the cut-off frequency value was a two-pass calculation of dynamic amplification factor, described in [36]. Filtering was applied to obtain maximum (quasi) static responses of the structure under calibration vehicles, later compared with the modelled static responses of the FE model.

The highest dynamic component resulted for the lightest vehicle V1. This is in line with the known phenomenon of the decreasing dynamic amplification factor with increasing gross vehicle weight [36,37,38,39,40]. The minimum, mean, and maximum velocity of all calibration vehicle passages in lane L1 was 67.9, 76.5, and 83.7 km/h, respectively.

The maximum value of the filtered time domain signal is marked with a white marker. These maximum values were calculated for all sensors and all passages, as shown in Figure 13b, Figure 14b and Figure 15b. The maximum values of the measurements on the same girder were averaged. Finally, one mean and one standard deviation value per girder was calculated and used in further studies, as shown in Figure 13c, Figure 14c, and Figure 15c.

The mean value (*μ),* standard deviation (*σ*), and coefficient of variation (CV) for all calibration vehicle passages are collected in Table 2. Passages in lane L1 cause approximately symmetric superstructure response, i.e., values on the external girders MG1 and MG4 and values on the internal girders MG2 and MG3 are similar. However, a closer look at the mean values reveals that vehicle V1 induced higher strains in the girder MG3 than in girder MG2, vehicle V2 induced approximately the same response in both girders, and vehicle V3 induced higher strains in girder MG2 than in girder MG3. Calibration vehicle drivers were told to drive as close to the lane centreline as possible. However, comparing the photos with the strain responses revealed that V2 and V3, on average, drove 5 and 10 cm from the centreline towards MG1, respectively. This deviation from the centreline was considered in all further studies.

### 2.3. Structural System and FE Model of the Considered Span

The left Ravbarkomanda viaduct has 15 spans, and the right one has 17. Both carry two lanes and a hard shoulder, and both superstructures are built from precast elements. The FEM study considers the span P14D of the right viaduct, which is separated from the neighbouring P13D by a finger-type expansion joint and continuously connected to the P15D through the slab.

**Figure 16:**
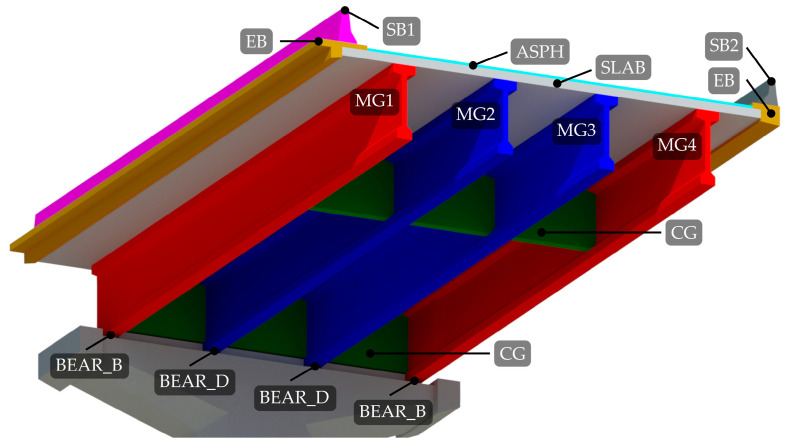
A render of a 3D section cut view of the span P14D with notations of the structural elements and elastomeric bearings, the same colour denoting the same material.

Figure 16 shows a 3D section render cut near the mid-span with abbreviations of the structural elements used throughout the paper. Elements with the same material characteristics have the same colour. Bearings BEAR_A and BEAR_C are not shown in Figure 16 due to the limited display. On the opposite side of the BEAR_B, the exterior main girders MG1 and MG4 are supported by BEAR_A. Similarly, on the opposite side of the BEAR_D, the exterior main girders MG2 and MG3 are supported by BEAR_C.

The structural system is based on precast I girders. The load from the road surface is transferred through the 8 cm asphalt and waterproofing layer (ASPH) to the 25 cm slab (SLAB). The bottom part of the slab is made of precast concrete segments, and the upper part with cast-in-place concrete, which makes the slab integral. The slab, except at the expansion joints, which divide the structure into units, runs continuously over the piers. From the slab, the load goes to the precast longitudinal (main) girders MG1 to MG4, connected in the transverse direction with prestressed concrete cross girders (CG) to form the grillage. Main girders run approximately from the centre of one pier to the centre of another pier and are not continuous over piers. Finally, the load is transmitted to the piers through the elastomeric bearings (BEAR_A, BEAR_B, BEAR_C, BEAR_D).

**Figure 17:**
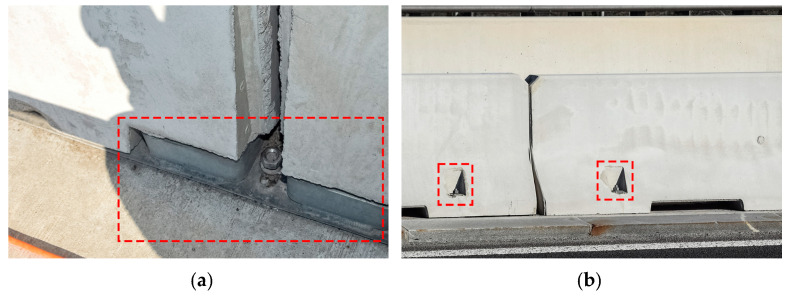
Anchoring detail (marked with red dashed line) of the (**a**) SB1 to the superstructure via anchorage plate, and (**b**) SB2 to the superstructure via anchors.

Concrete safety barrier SB1 is (partially) anchored to the cast-in-situ edge beam (EB) through anchoring plates (Figure 17a) and SB2 through the anchors (Figure 17b), as described in detail in Section 2.4.2.

**Figure 18:**
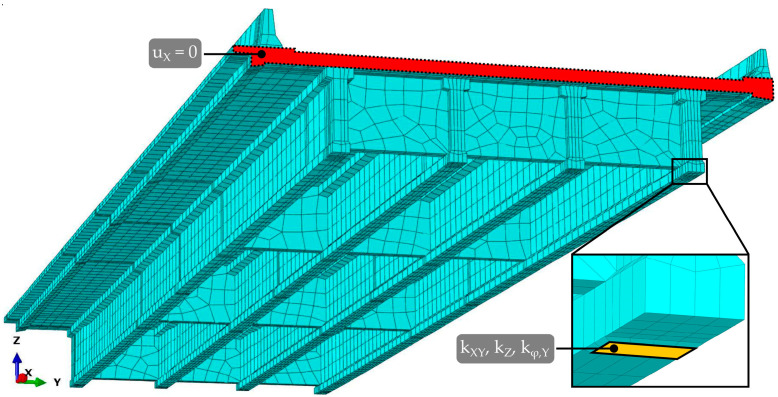
The FE model in Abaqus CAE 2016 FEA with the labelled constrained area (red surface) and detailed view of the viaduct bearings area (yellow surface).

The complex geometry was created in AutoCAD 2023 [41] software as a 3D solid, exported as ACIS (.sat) geometry file type and imported in the Abaqus CAE 2016 [42] finite element analysis (FEA) software for further analyses. The span geometry followed the design documentation [43], with minor simplifications of the edge beam. All finite elements were of hexahedral shape, as shown in Figure 18. Before running the extensive FE analyses, a mesh convergence study of FE types and sizes was performed. For bending problems, the study confirmed the superior behaviour of 20 node quadratic (C3D20R) elements compared to linear (C3D8R) elements, where the 0.1 m global element mesh size produced comparable results as five times bigger C3D20R elements. Therefore, a model of approximately 0.5 m global size C3D20R elements was selected for all further studies, resulting in roughly 20.000 finite elements for the entire span.

The slab runs continuously from P14D to P15D. In the FE model, this continuity was considered by preventing displacements in the longitudinal (X) direction along the entire red area of the slab and asphalt layer, as shown in Figure 18. Longitudinal displacements on the actual structure are prevented by a series of elastomeric bearings in span P15D, P16D, and P17D (4th unit in Figure 3). No connection constraints were considered on the other side of the span, with P14D and P13D separated by a finger-type expansion joint. 

Elastomeric bearings were modelled as ‘Cartesian + Rotation’ connector sections, with translational stiffness (k_XY_) in X and Y directions, vertical stiffness (k_Z_) in Z direction, and rotational stiffness (k_φ,Y_) around the Y axis. The connector section was assigned through the ‘Wire’ element to the reference point in the bearing-girder contact surface (shown in Figure 18 with yellow colour), connected with other points on this surface with constraints. This enabled to support of the whole bearing area by springs.

All eight structural elements were considered with elastic isotropic material, according to the design documentation [43,44] presented in Table 3. The design properties of the elastomeric bearings are given in Table 4. The specific weight of all structural elements was not optimised. A concrete elements value of 25 kN/m^3^ and an asphalt layer value of 25.83 kN/m^3^ was considered, according to [43,44].

### 2.4. Model Updating

#### 2.4.1. Objective Function

The objective function, also called the “index of discrepancy”, is a function that combines measured and modelled responses. The goal of the FE model updating is to reduce the difference between those responses, and the role of the objective function is to formulate a problem where its minima best matches the model and actual structure. Various authors proposed different FE model updating objective functions [12]. This study considered the sum of squared relative differences with standard deviation as a normalisation term. This function was modified by averaging the measured values on the nearby sensors, for example, SG_02-1, SG_02-2, and SG_02-3 (Figure 12). Those values then corresponded to the SG_02 sensor. The reason to compare groups instead of individual sensors was to reduce the errors due to inadequate determined micro-location and faulty behaviour of the individual sensors.

Similarly, the modelled values on the nearby sensors were averaged. One should note that although SG_01, SG_02, SG_03, and SG_04 were only a notation for average values on the strain gauge sensors on the MG1, MG2, MG3, and MG4, respectively, they were referred to throughout the entire paper as sensors. The objective function was defined as follows:

(1) $$J=\overset{n_{v}}{\underset{v=1}{\sum }}\overset{n_{g}}{\underset{g=1}{\sum }}\frac{{\left(z_{\mathrm{num},v,g}-z_{\exp,v,g}\right)}^{2}}{\;\sigma_{\exp,v,g}{}^{2}}$$

where $z_{\mathrm{num},v,g}$ and $z_{\exp,v,g}$ were calculated as,

(2) $$z_{\mathrm{num},v,g}=\frac{1}{n_{g,s}}\sum_{s=1}^{n_{g,s}}\varepsilon_{\mathrm{num},v,g,s}\;\mathrm{and}$$

(3) $$z_{\exp,v,g}=\frac{1}{n_{g,s}}\sum_{s=1}^{n_{g,s}}\left(\frac{1}{n_{v,p}}\sum_{p=1}^{n_{v,p}}\varepsilon_{\exp,v,g,s,p}\right)$$

- v denotes the calibration vehicle index;
- $n_{v}$ denotes the number of calibration vehicles considered (3 in this study);
- g denotes the main girder index;
- $n_{g}$ denotes the number of main girders considered (4 in this study);
- $\;\sigma_{\exp,v,g}$ denotes the standard deviation of measured strains for main girder *g* and vehicle *v;*
- s denotes the strain gauge sensor index on the selected main girder;
- $n_{g,s}$ denotes the number of strain gauges considered in a given girder *g* (2 or 3 in this study);
- p denotes the passage index of the selected calibration vehicle;
- $n_{v,p}$ denotes the number of vehicle *v* passages;
- $\varepsilon_{\mathrm{num},v,g,s}$ denotes the FE model longitudinal strain, oriented parallel to the X (longitudinal) direction of the viaduct—$\varepsilon_{XX}$, (see Figure 18) in the selected node that corresponds to the s-th strain gauge sensor on the g-th main girder, caused by the v-th calibration vehicle positioned on location that results in the maximum strain at sensors SG_0*g*;
- $\varepsilon_{\exp,v,g,s,p}$ denotes the maximum measured longitudinal strain (Section 2.2.2) in the s-th strain gauge sensor on the g-th main girder, caused by the v-th calibration vehicle during p-th passage.

#### 2.4.2. Manual FE Model Updating

A nonlinear optimisation requires an initial manual FE model calibration based on engineering judgment and the agreement between the FEA and measurements. Lachinger et al. [45] reported the importance of manual updating, where the objective function value was reduced by 45%, while nonlinear optimisation that followed reduced the objective function only by an additional 10%.

To minimise the modelling errors, to more realistically model the boundary conditions, and to observe the influence of non-structural elements, it was decided to use the 3D solid finite elements. Elastomeric bearings were modelled as springs, as described in Section 2.3. Concrete safety barriers were modelled as structural elements. The contribution of the safety barriers to the superstructure bending stiffness is complex, mainly due to the type of anchorage to the viaduct deck. If safety barriers were only laid on the superstructure, their contribution would be negligible. However, safety barriers are attached to the superstructure with anchorage plates or directly by anchors, as shown in Figure 17. Such interaction is challenging to capture by strain gauge sensors alone. However, it was expected that the manual FE model updating with the anchorage reduction factors $\varphi_{\mathrm{SB}1}$ and $\varphi_{\mathrm{SB}2}$ would address it in a simplified way. Six different FE models were created, each of them with a different value of anchorage reduction factors $\varphi_{\mathrm{SB}1}$ and $\varphi_{\mathrm{SB}2}$ for safety barriers 1 and 2 (SB1 and SB2, according to Figure 16). With the anchorage reduction factor, Young’s modulus of the safety barriers was multiplied to reduce their contribution to the global stiffness of the superstructure. Values of the $\varphi_{\mathrm{SB}1}$ and $\varphi_{\mathrm{SB}2}$ for SB1 and SB2 are written in the brackets beside the model names, respectively:

- M1 (1.0, 1.0);
- M2 (0.5, 0.5);
- M3 (0.001 ≈ 0, 0.001 ≈ 0);
- M4 (0.5, 1.0);
- M5 (0.001 ≈ 0, 1.0);
- M6 (0.001 ≈ 0, 0.5).

The M1 model represented the full connection of both barriers with the superstructure, and the M3 model represented nearly no interaction of both barriers. In model M1, it was assumed that although the 6 m barriers are anchored and longitudinally separated, their behaviour was as if they were monolithically connected to the superstructure. This assumption was also considered in a previous study [46], where the numerical evaluation of the dynamic characteristic of the Ravbarkomanda viaduct was performed. Other models considered the variations of M1 and M3. M4, M5, and M6 interacted less with SB1 than SB2 because the latter are anchored (Figure 17a), and the SB1 is laid on the anchoring plates (Figure 17b). It was expected that SB1 would contribute less to the bending stiffness of the superstructure than SB2.

#### 2.4.3. Automatic Nonlinear Optimisation

Nonlinear optimisation is performed with optimisation algorithms. Many were developed even before the electronic computer era, but modern computers significantly accelerated the development of new algorithms [47]. Often-used optimisation algorithms in the civil engineering field are first-order, second-order, direct, and population methods. Besides single-objective optimisation, as is the case in this paper, algorithms are also developed for multi-objective optimisation, where optimisation is performed simultaneously with respect to several objectives, as in [48]. Since not all optimisation algorithms always produce optimal results and based on the previous experiences and similar studies from the literature, it was decided to benchmark the performance of the following algorithms:

- Sequential Least SQuares Programming (SLSQP) [49],
- Particle Swarm Optimisation (PSO) [50], and
- Genetic Algorithm (GA) [51].

While sequential quadratic programming methods were inspired by Newton’s method for solving systems of nonlinear equations [49], the PSO and GA methods are population-based methods. PSO was inspired by animal behaviours, for example, by a bird, which “swarms” randomly through the search space, recording and communicating with other birds about the best solution they have discovered [11]. GA was developed based on biological evolution, where fitter individuals are more likely to pass on their genes to the next generation. An individual’s fitness for reproduction is inversely related to the value of the objective function at that point [47].

When performing the nonlinear optimisation of the FE model, it is desirable that the FEA software can interact with the external programming platform such as MATLAB, Python, and Mathematica, where input files for the analysis are prepared, the FEA job submitted, the FEA results (output files) are checked, and the new input files prepared based on the optimisation algorithm decision. On the other hand, some FEAs, like Ansys [52], already include optimisation modules. In this study, Abaqus CAE 2016 [42] FEA software was used, with Python 3.7 utilising scipy.optimise.minimise [53] and pymoo [51] libraries.

## 3. Results

### 3.1. Sensitivity Study

**Figure 19:**
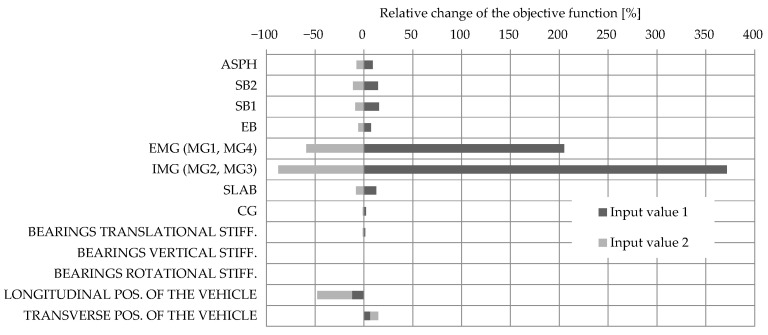
The sensitivity study results with the influence of structural elements Young’s modulus, bearing stiffness, and the position of the vehicle V1 on the value of the objective function.

The results of the deterministic sensitivity analysis provided information to understand the impact of individual structural elements’ stiffness and calibration vehicle position on the values of the objective function and, thus, guided the next stage of the model updating process. Only vehicle V1 in lane L1, applied as a series of concentrated loads, was considered in this study, and the M1 FE model with design properties was used. Figure 19 presents the relative change of the objective function value due to the selected bottom and upper values, denoted as input values 1 and 2, respectively. These values varied by 25% for all cases compared to the design values. Table 5 additionally clarifies the properties of the modified variables.

The results showed that the external (EMG) and internal (IMG) main girders most significantly impact the relative change of the objective function. Overall, −25% variation in the stiffness of the EMG and IMG had 205% and 372% impacts on the objective function value. This was expected since those elements are designed to carry most of the traffic load, and measurements were performed on those elements. The third and fourth elements with the greatest impact were safety barriers SB1 and SB2. Further, ±25% variation in the stiffness of the SB1 and SB2 had less than ±15% impact on the objective function value. For the asphalt layer, slab, and edge beam, ±25% variation in the stiffness had less than ± 13% impact on the objective function value. The negligible impact had cross girders and viaduct bearings. Based on this, the elements were arranged into important (group “EMG and IMG elements”) and less important (group “other elements”) groups. Consequently, the number of variables for automatic nonlinear optimisation was reduced from 13 to 2: Young’s modulus adjustment factor of the group “EMG and IMG elements” ($\alpha_{\mathrm{MG}}$) and Young’s modulus adjustment factor of group “other elements” ($\alpha_{\mathrm{OTHER}}$). Element grouping was not reflected by the same Young’s module for all elements in the group but by varying their initial (design) modulus during the updating process for equal relative value—the adjustment factor.

In addition to the significant impact of the main girders, the vehicle’s longitudinal position notably affected the objective function value. In fact, 1 m error in its estimated position changed the objective function by approximately 35%, which has greater impact than ± 25% variation of the SB1s or SB2s Young’s modulus. Finding the exact position of the calibration vehicle that caused the greatest response was significant in this study, where model updating was based on maximum strain measurements under full-speed calibration vehicle passages. Unlike during the diagnostic or proof load tests, where the vehicles are applied stationary at the predefined location, the exact position of the calibration vehicle was unknown. Therefore, a separate analysis was performed for all three calibration vehicles to find the longitudinal position of the vehicle that caused the greatest strains in the SG_01, SG_02, SG_03, and SG_04 sensors and, consequently, in the objective function (Figure 12). Again, these sensors represent the average value of two or three nearby strain gauge sensors on the same girders.

**Figure 20:**
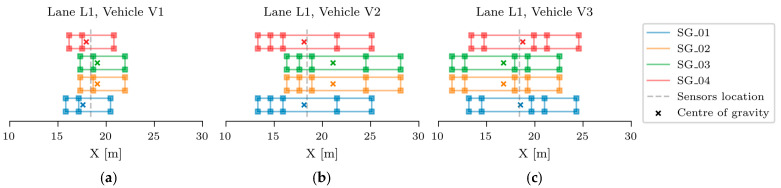
Position of the calibration vehicles V1 (**a**), V2 (**b**), V3 (**c**), that cause the greatest response in sensors SG_01,SG_02, SG_03 and SG_04.

Figure 20 presents the results of this longitudinal position study for Vehicles V1, V2, and V3. Blue, orange, green, and red colours denote the positions of the calibration vehicles that cause the greatest response in sensors SG_01, SG_02, SG_03, and SG_04. The crosses within each vehicle denote their centres of gravity, and the dashed grey lines indicate the approximate location of the sensors in the span. The maximum responses in the IMG elements (SG_02 and SG_03) result from vehicles placed in one position, and the maximum responses in the EMG elements (SG_01 and SG_04) result from vehicles placed in another longitudinal position. Longitudinal strains under the driving lane vehicles (lane L1) were approximately 30–40% greater on the internal IMG elements than on the EMG elements. 

For this reason and to reduce the number of FE model updating analyses, it was concluded to position the first axle of the vehicles in the following analyses based on the maximum response in sensors SG_02 and SG_03, which was at X = 21.95 m, 28.15 m, and 22.55 m, for V1, V2, and V3, respectively. An additional sensitivity study of vehicle transverse position, which is beyond the scope of this paper, also emphasised the importance of the exact vehicle transverse position. Even the 0.1 m variation of the calibration vehicles’ transverse position had a non-negligible influence on the objective function. Therefore, an accurate estimate of the transverse position, as described in Section 2.2.2, is essential.

### 3.2. Updated FE Model

A two-stage, manual and automatic FE model updating was performed in the first study. First, six FE models (M1-M6, described in Section 2.4.2) were created in the manual updating stage. The main differences among models involved different safety barriers’ contribution to the superstructure’s stiffness, accounted for by the anchorage reduction factors $\varphi_{\mathrm{SB}1}$ and $\varphi_{\mathrm{SB}2}$. After that, two comprehensive model updating studies of manually refined FE models were performed.

**Figure 21:**
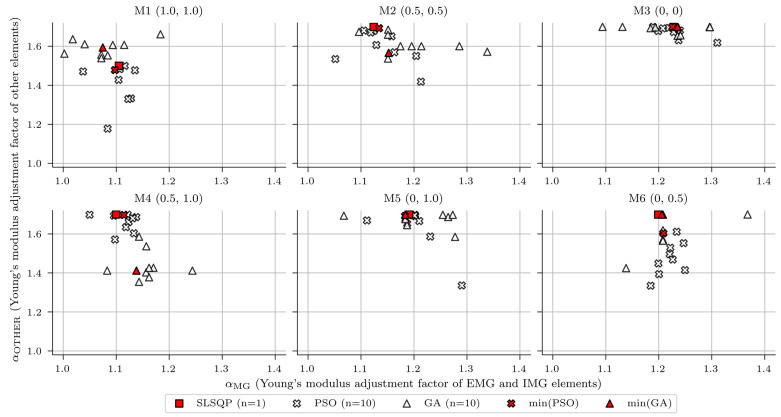
Results of the nonlinear optimisation for six different finite element (FE) models, three different optimisation algorithms, and the following two variables: $\alpha_{\mathrm{MG}}$ and $\alpha_{\mathrm{OTHER}}$.

In the first study, the automatic nonlinear optimisation utilised three different nonlinear optimisation algorithms (SLSQP; PSO and GA, Section 2.4.3) for two variables (Section 3.1) and one objective function (Section 2.4.1). Figure 21 summarises the nonlinear optimisation results. The horizontal axis displays the updated values of the variable $\alpha_{\mathrm{MG}}$ (Young’s modulus adjustment factor of EMG and IMG elements). Similarly, the vertical axis shows updated values for $\alpha_{\mathrm{OTHER}}$ (Young’s modulus adjustment factor of other elements). The plane where algorithms searched for the optimal solution was bounded by the minimum and maximum values of 1.0 and 1.7 for $\alpha_{\mathrm{MG}}$ and $\alpha_{\mathrm{OTHER}}$. Some initial calculations were devoted to selecting and narrowing this range iteratively. A lower bound value of 1.0 was chosen based on the sensitivity analysis (Figure 19), which suggested to increase the design stiffness of all structural elements to minimise the objective function. Other parameters of the SLSQP algorithm besides bounds, which should not be mistaken with updating parameters (variables), were *x0* (initial guess, value of 1.0) for both variables to be updated, *ftol* (precision goal for the objective function value in the stopping criterion, value of 1 × 10^−5^), and *eps* (step size for the numerical approximation of the Jacobian, value of 1 × 10^−2^). Other parameters for both PSO and GA algorithms beside bounds were *pop_size* (population size) of 10 and 10 generations as termination criterion, representing overall 100 evaluations per individual PSO or GA analysis.

The red square marker in Figure 21 represents the values of both variables for the last evaluation, given by the SLSQP algorithm. The white cross and triangular markers represent values of both variables for all 10 evaluations within the last (10th) population for PSO and GA algorithms, respectively. The red cross and triangular markers represent values of both variables that correspond to the minimum value of the objective function within the last (10th) population, respectively. Their numerical values are given in Table 6.

**Figure 22:**
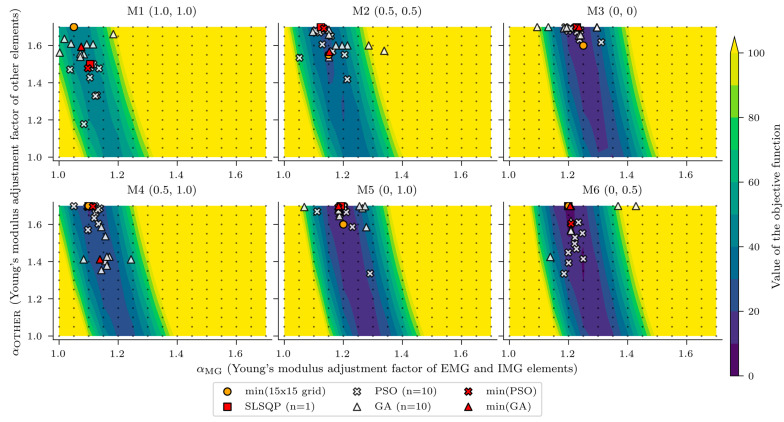
Values of the objective functions, calculated for 225 different input combinations of variables $\alpha_{\mathrm{MG}}$ and $\alpha_{\mathrm{OTHER}}$ (shown by contours) and results of the nonlinear optimisation.

The updated variables from three algorithms give different results. The background for such outcomes was examined in more detail by calculating the objective function values for different values of both variables. The objective function of each model was calculated for 15 different linearly spaced values of variables $\alpha_{\mathrm{MG}}$ and $\alpha_{\mathrm{OTHER}}$, both between values of 1.0 and 1.7. Overall, the objective function was calculated for 15 × 15 = 225 different combinations of $\alpha_{\mathrm{MG}}$ and $\alpha_{\mathrm{OTHER}}$. Each black dot in Figure 22 represents the value of both variables where the objective function was calculated.

Contours of the objective function, shown in Figure 22, revealed the insensitivity of the ratio of updated to design Young’s modulus of other elements to the objective function, since the shape around minimum values of the objective functions resembles a riverbed or valley rather than a bowl. However, $\alpha_{\mathrm{OTHER}}$ still has some influence on the objective function since the contours are not straight vertical lines. The objective function contours for models M1, M4, and M6 also show that the minimum of the objective function corresponds to the maximum value of the variable $\alpha_{\mathrm{OTHER}}$, which is 1.70. This value is far too high and could not represent the value that best fits the measured response. For example, for the slab, which belongs to the “other elements” group, this would increase the Young’s modulus to 1.7 × 33 GPa = 56.1 GPa. Similarly, models M2, M3, and M5 overestimate the values of variable $\alpha_{\mathrm{MG}}$, all of them greater than 1.5. 

**Figure 23:**
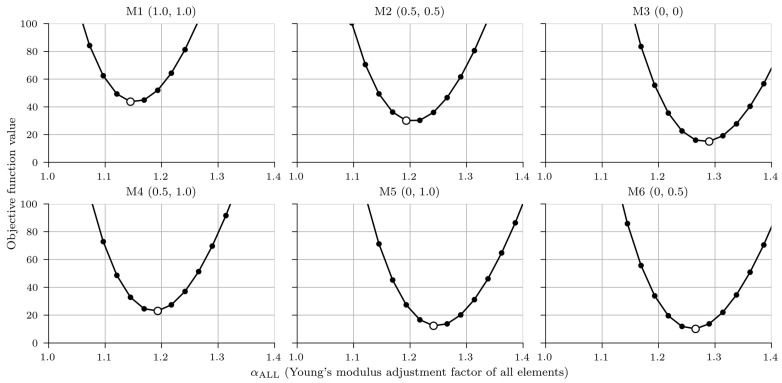
Objective functions for six different FE models with a white marker denoting the minimum value.

To overcome challenges with two variables, it was decided to combine variables $\alpha_{\mathrm{MG}}$ and $\alpha_{\mathrm{OTHER}}$ into one. The model was a function of only one variable—Young’s modulus adjustment factor of all elements ($\alpha_{\mathrm{ALL}}$)—which could also be interpreted as a global stiffness increase factor. By doing so, it was faster to calculate the objective function for each FE model for 30 linearly spaced values of the $\alpha_{\mathrm{ALL}}$, between values of 1.0 and 1.7 than to perform the nonlinear optimization to find the minimum value of the objective function. Minimum objective function values and corresponding variables $\alpha_{\mathrm{ALL}}$ for six different FE models are shown in Figure 23 and Table 7.

$\alpha_{\mathrm{ALL}}$ The M6 model best fit the measured response. It had anchorage reduction factors $\varphi_{\mathrm{SB}1}$ and $\varphi_{\mathrm{SB}2}$ values of 0 and 0.5, respectively, and the updated Young’s modulus adjustment factor of all elements $\alpha_{\mathrm{ALL}}$ equal to 1.27. In other words, to best fit the measured response under the calibration vehicles, the initial FE model had to be stiffened by multiplying the design Young’s modulus of all elements by 1.27, except of the safety barriers SB1 and SB2, which should have design value of Young’s modulus multiplied by 0 and 0.50 × 1.27 = 0.64, respectively. The second and third best-fit models are M5 and M3, with $\alpha_{\mathrm{ALL}}$ value of 1.24 and 1.29, respectively.

**Figure 24:**
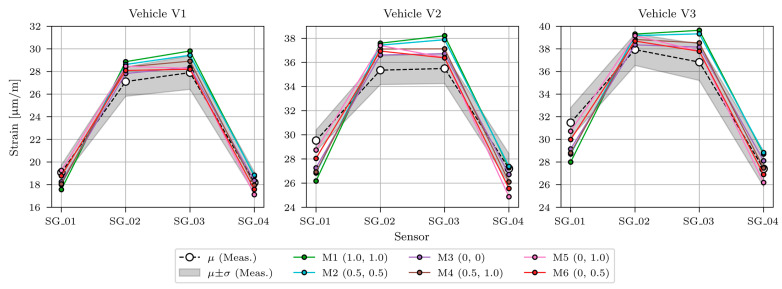
Comparison of maximum strains from measurements and updated FE model under calibration vehicles V1, V2, and V3 for all six manually refined FE models.

Figure 24 shows measured and modelled maximum strains under calibration vehicles V1, V2, and V3 for sensors SG_01, SG_02, SG_03, and SG_04. Measured strains (same values as in Figure 13, Figure 14 and Figure 15) are shown as mean (*μ*) and standard deviation (*σ*) values. Strains of the best-fit model M6 are shown with the red line.

In the last analysis, a nonlinear optimisation of the initial FE model was performed for the following three variables:

- $\alpha_{\mathrm{ALL}}$ (Young’s modulus adjustment factor of all elements);
- $\varphi_{\mathrm{SB}1}\;$(SB1 anchorage reduction factor);
- $\varphi_{\mathrm{SB}2}\;$(SB2 anchorage reduction factor).

In this analysis, the variables $\varphi_{\mathrm{SB}1}$ and $\varphi_{\mathrm{SB}2}$, set manually in the previous studies, were part of the automatic optimisation together with variables $\alpha_{\mathrm{ALL}}$. While it was concluded from the previous study that the M6 model with $\varphi_{\mathrm{SB}1}=0$ and $\varphi_{\mathrm{SB}2}=0.5$ is the best-fitting model, the goal of this study was to find values of $\varphi_{\mathrm{SB}1}$ and $\varphi_{\mathrm{SB}2}\;$(and $\alpha_{\mathrm{ALL}}$) that fit the actual structure even better than the M6 model. All three optimisation algorithms were defined similarly to the previous study, where only two variables were updated. The variable bounds were set from 1.0 to 1.7 for the $\alpha_{\mathrm{ALL}}$, from 0.001 to 1.0 for the $\varphi_{\mathrm{SB}1},$ and from 0.001 to 1.0 for $\varphi_{\mathrm{SB}2}$. Based on the previous results, it was expected that the updated FE model would be between models M6 and M5. This was confirmed (Table 8). It can be concluded that the impact of SB1 is nearly negligible, while the stiffness of SB2 should be considered.

**Figure 25:**
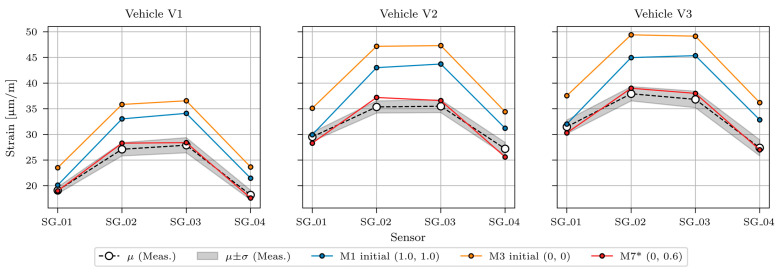
Comparison of maximum strains from measurements, initial FE models (M1 initial, M3 initial), and best-fit updated FE model (M7*) under calibration vehicles V1, V2, and V3.

The SLSQP algorithm found the minimum value of the objective function with a value of 10.23, achieved when the Young’s modulus of the ASPH, EB, SLAB, CG, EMG, and IMG elements was 125% of their design values. SB1 stiffness had a negligible effect on the response of the superstructure under calibration vehicles (its Young’s modulus was 0.00 × 125% = 0.00% of its design value), and SB2 Young’s modulus was 0.56 × 125% = 70% of its design value. The best fit FE model with such properties is denoted in the following as “M7* model”. Figure 25 shows the comparison of measured, initial (M1 initial, M3 initial) and updated FE model M7* under calibration vehicles V1, V2, and V3. Strains of the initial model M1 (M1 initial) and M3 (M3 initial) are shown in blue and orange, respectively. They demonstrate how material characteristics taken from the design documentation can overestimate the strains.

All final values of updated variables were within the expected range. The 1.25 increase in bending stiffness of all structural elements may seem high. However, it can be justified by a large amount of prestressing tendons and other reinforcing steel, primarily in the precast girders. Calculated effective geometrical properties, including the contribution of the prestressing tendons not presented in this paper, increased the stiffness by a factor of 1.10. An additional growth by a factor of 1.05 to 1.10 can result from increased Young’s modulus with time, the case study viaduct being over 50 years old. Finger-type expansion joint between span P14D and P15D also increased the stiffness, as well as Young’s modulus dependency on the loading rate, which was not quasi-static due to the full speed of the calibration vehicles. Variable $\varphi_{\mathrm{SB}2}$ was expected to be higher compared to $\varphi_{\mathrm{SB}1}$, since the safety barrier SB2 is attached to the superstructure directly with anchorages, thus being more connected to the superstructure than the safety barrier SB1, anchored indirectly via anchorage plates.

It is vital to understand that the contribution of the safety barriers to the bending stiffness of the superstructure should be considered with care, especially in the limit states, since the viaduct was not tested for such high load levels. Examination of the load-dependent contribution of safety barriers and other (non-structural) elements to the superstructure stiffness will be possible in future studies, where the FE model updating would include random B-WIM weighed vehicles of different load levels, exceptional transport vehicles being the most interesting. Another future study that could bridge the gap between the FE model updating based on static SHM measurement and dynamic acceleration-based SHM measurements could update the model based on identified modal parameters and comparison with the results from the (quasi) static measurements presented in this paper. 

## 4. Conclusions

B-WIM systems measure axle loads of most passing vehicles. However, despite capturing bridge strains under the crossing vehicles, this information has not been used to monitor structural performance. This paper presents a model updating concept using data from B-WIM sensors. The study focused on a response of a multi-span precast girder-type viaduct with a B-WIM installed as a part of the long-term monitoring system. Longitudinal (bending) strain records in the time domain induced by calibration vehicles were filtered and statistically processed. Their maximum values were used to update the FE model.

The initial FE model was based on the design values of material and geometrical properties and was manually refined. Six different FE models were built to study the complex safety barrier–superstructure interaction, considered through anchorage reduction factors, one for each safety barrier. Automatic model updating of all six FE models utilised three nonlinear optimisation algorithms and two variables determined by a sensitivity analysis, Young’s modulus of main girders, and all other elements. The objective function was calculated for each FE model for different values of both input variables. 

In the following FEM study, each of the six FE models was tuned by a single variable ($\alpha_{\mathrm{ALL}}$), representing the Young’s modulus adjustment factor for all structural elements. The model with $\alpha_{\mathrm{ALL}}$, $\varphi_{\mathrm{SB}1},$ and $\varphi_{\mathrm{SB}2}$ values of 1.27, 0, and 0.50, respectively, best described the actual structure. Finally, a similar concept was applied to update $\alpha_{\mathrm{ALL}}$, $\varphi_{\mathrm{SB}1},$ and $\varphi_{\mathrm{SB}2}$ automatically. The optimal match was obtained when increasing the Young’s modulus of all structural elements to 125% of their design values. Safety barrier SB1 contribution to the overall bending stiffness was found negligible, and SB2 contribution was best encountered with 70% of Young’s modulus design value. The analysis confirmed that an initial manual FE model updating study was needed to correctly consider the non-structural elements during the automatic nonlinear optimisation. The FE model, which strictly followed the design documentation, neglected the influence of safety barriers and therefore overestimated the design stiffness of structural elements by 4%, compared to the final best-fitting M7* model, where the influence of the safety barriers was considered.

The pseudo-static B-WIM loading data were successfully used during the model updating process. The future aim is to extend this approach to using random B-WIM-weighed vehicles for FE model updating and long-term monitoring of structural parameters and load-dependent phenomena. Such a monitoring framework also has the potential to integrate dynamic and static SHM monitoring issues.

## Figures and Tables

**Table 1:** Axle loads, axle spacing, and Gross Vehicle Weight (GVW) of the calibration vehicles.

	1st Axle		2nd Axle		3rd Axle		4th Axle		5th Axle	
Vehicle	Load [kN]	Spacing [m]	Load [kN]	Spacing [m]	Load [kN]	Spacing [m]	Load [kN]	Spacing [m]	Load [kN]	GVW [kN]
V1	67.69	3.30	85.35	1.35	88.29	/	/	/	/	241.33
V2	68.67	3.60	93.20	5.60	76.52	1.30	75.54	1.30	76.52	390.44
V3	68.67	3.30	87.31	1.35	87.31	5.17	76.52	1.33	76.52	396.32

**Table 2:** The number of signals (*n*), mean (*μ)*, standard deviation (*σ*), and coefficient of variation (CV) for maximum measured values of calibration vehicle passages in lane L1.

*n, μ* [μm/m], *σ* [μm/m], CV [%]		V1	V2	V3
SG_01	*n*	32	34	36
	*μ*	19.1	29.5	31.5
	*σ*	0.7	0.9	1.3
	CV	3.5	2.9	4.2
SG_02	*n*	48	51	54
	*μ*	27.1	35.4	37.9
	*σ*	1.3	1.2	1.4
	CV	4.8	3.4	3.7
SG_03	*n*	48	51	54
	*μ*	27.9	35.5	36.8
	*σ*	1.5	1.3	1.6
	CV	5.3	3.5	4.4
SG_04	*n*	48	51	54
	*μ*	18.2	27.2	27.4
	*σ*	0.9	1.2	1.6
	CV	5.2	4.6	5.7

**Table 3:** Material properties of structural elements according to design documentation [43,44].

Element	Abbreviation	Young’s Modulus [GPa]	Poisson Ratio
Slab	SLAB	33	0.20
External main girder	EMG (MG1, MG4)	35	0.20
Internal main girder	IMG (MG2, MG3)	34	0.20
Cross girder	CG	35	0.20
Safety barrier 1	SB1	33	0.20
Safety barrier 2	SB2	33	0.20
Edge beam	EB	33	0.20
Asphalt	ASPH	8	0.35

**Table 4:** Properties of elastomeric bearings according to design documentation [43].

Element	Abbreviation	Translational Stiffness [kN/m]	Vertical Stiffness [kN/m]	Rotational Stiffness [kNm]
Bearing type “A”	BEAR_A	3.10 × 10^3^	1.08 × 10^6^	3.09 × 10^3^
Bearing type “B”	BEAR_B	2.43 × 10^3^	8.43 × 10^5^	2.32 × 10^3^
Bearing type “C”	BEAR_C	3.72 × 10^3^	1.56 × 10^6^	7.32 × 10^3^
Bearing type “D”	BEAR_D	2.92 × 10^3^	1.22 × 10^6^	5.49 × 10^3^

**Table 5:** List of variables considered in the sensitivity analysis with the description of modified variables.

Element/Variable/Property	Input Value 1 ^1^	Input Value 2 ^1^	Description
ASPH, SB1, SB2, EB, EMG (MG1, MG4), IMG (MG2, MG3), SLAB, CG	0.75 × design	1.25 × design	Young’s modulus change
BEARINGS TRANSL. STIFF.	0.75 × design	1.25 × design	Horizontal (X and Y) stiffness change
BEARINGS VERT. STIFF.	0.75 × design	1.25 × design	Vertical (Z) stiffness change
BEARINGS ROT. STIFF.	0.75 × design	1.25 × design	Rot. (around Y) stiffness change
LONGIT. POS. OF THE VEHICLE ^2^	21.95 m – 1 m	21.95 m + 1 m	Longitudinal position change
TRANSV. POS. OF THE VEHICLE ^3^	3.77 m – 0.1 m	3.77 m + 0.1 m	Transverse position change

^1^ Design values from Table 3 and Table 4. ^2^ Longitudinal position of the first wheel relative to the coordinate system. ^3^ Transverse position of the wheel, which is closer to the SB1, relative to the coordinate system.

**Table 6:** Ratios of updated Young’s modulus after nonlinear optimisation to design values for six different FE models, three optimisation algorithms, and two variables.

Model	SLSQP		PSO		GA	
	EMG and IMG Elements	Other Elements	EMG and IMG Elements	Other Elements	EMG and IMG Elements	Other Elements
M1	1.10	1.50	1.10	1.48	1.07	1.59
M2	1.12	1.70	1.13	1.69	1.15	1.57
M3	1.23	1.70	1.23	1.70	1.24	1.70
M4	1.10	1.70	1.11	1.70	1.14	1.41
M5	1.19	1.70	1.18	1.69	1.18	1.70
M6	1.20	1.70	1.21	1.60	1.21	1.70

**Table 7:** Minimum objective function values and values of the corresponding variables $\alpha_{\mathrm{ALL}}$ for six different FE models.

	M1	M2	M3	M4	M5	M6
The minimum objective function value	43.77	30.13	15.09	23.05	12.36	10.17
$\alpha_{\mathrm{ALL}}$	1.14	1.19	1.29	1.19	1.24	1.27

**Table 8:** Results of the nonlinear optimisation for three variables and three optimisation algorithms.

	SLSQP	PSO	GA
Value of the objective function	10.23	14.51	10.83
$\alpha_{\mathrm{ALL}}$ (Young’s modulus adjustment factor of all elements)	1.25	1.24	1.23
$\varphi_{\mathrm{SB}1}\;$(SB1 anchorage reduction factor)	0.00	0.12	0.19
$\varphi_{\mathrm{SB}2}\;$(SB2 anchorage reduction factor)	0.56	0.90	0.69
$\alpha_{\mathrm{ALL}}\cdot \varphi_{\mathrm{SB}1}$	0.00	0.15	0.23
$\alpha_{\mathrm{ALL}}\cdot \varphi_{\mathrm{SB}2}$	0.70	1.12	0.85

## Data Availability

The finite element models and the data from the case study are available upon request from the corresponding author.
